# Admission to intensive care: a matter of shared decision-making in the very old?

**DOI:** 10.1007/s41999-026-01520-z

**Published:** 2026-06-15

**Authors:** Gavin M. Joynt, Gabriele Leonie Schwarz

**Affiliations:** 1 Department of Anaesthesia and Intensive Care, The Chinese University of Hong Kong, Hong Kong SAR, China; 2 Department of Surgical Services, Haukeland University Hospital, Bergen, Norway; 3 Department of Clinical Medicine, University of Bergen, Bergen, Norway

**Keywords:** Decision Making, Shared, Aged, 80 and over, Intensive Care, Triage, Autonomy, Personal, Ageism

## Abstract

Intensive Care Unit (ICU) admission for very old patients involves complex, critical, and time-sensitive decision-making. In the very old, the chances of a satisfactory functional recovery are more limited, remaining life years are fewer, and the quality of life after ICU discharge is often reduced. Predicting outcome, ICU length of stay, and establishing a burden-benefit balance is clinically challenging. Understanding the value that very old patients place on functional outcomes and quality of life is paramount. However, the very old are often exposed to over-servicing and overly aggressive treatments, under-servicing due to ageism, or receiving care that conflicts with their goals or best interests. Shared decision-making (SDM) is a collaborative process between healthcare professionals and patients or proxies, aligning with the high value that many modern societies place on individual autonomy. Quality SDM has the potential to integrate the treatment team's knowledge and recommendations with patient preferences, thereby improving the appropriateness of critical care provided. SDM can be supported by a time-limited trial of intensive care. However, when resources are scarce and patient admission must be restricted through a triage process, initial decision-making excludes SDM, as the principle of autonomy is set aside in favour of justice and the fair distribution of limited resources. Similarly, when admission is deemed futile or care is potentially inappropriate, SDM is unsuitable. Avoiding ageism in this context is challenging and requires carefully considering the effects and biases of chronological and biological age on prognosis and triage decisions.

## Key summary points

**Aim:**

To review the justification and appropriate use for SDM at the time of ICU admission.

**Findings:**

By promoting patient autonomy, SDM is a useful tool for integrating the very old patient’s values and preferences into ICU admission decisions. Patient autonomy and SDM must be subordinated to other principles and approaches in the very old when resource limitations, futility, or potentially inappropriate treatment determine the outcomes of admission decisions.

**Message:**

When used correctly, SDM helps stakeholders making appropriate ICU admission decisions.

## Introduction

Admitting a patient to an Intensive Care Unit (ICU) involves multidimensional, critical, and time-sensitive decisions, primarily based on the patient’s potential for recovery from organ dysfunction or the need for advanced monitoring to prevent organ failure (Table 1). The main moral principles supporting ICU admission are patient beneficence and non-maleficence, that is, maximising therapeutic benefit and minimising the side-effects of treatments, including the potential health risks of denying ICU admission. Patients generally have the overriding right to accept or decline any offered ICU admission, exercising their moral right to autonomy, although this right may not be absolute in certain specific circumstances. In very old patients, in this context defined as 80 years or older and hereafter referred to as “older”, the decision becomes more complex because the potential for acceptable recovery is limited, remaining life years are relatively few, and quality of life after ICU discharge is often reduced. These factors may make ICU admission less desirable in advanced age, especially when chronological age is complicated by substantial co-morbidity or functional impairments.

**Table 1:** Multidimensional approach to ICU admission decisions in advanced age

Contextual factors	Religious and cultural expectations
	Legal and professional regulations
Prognostic factors	Reversibility of the index condition
	Illness severity of the index condition (extend and slope of multi-organ dysfunction)
	Baseline functional status
	Chronic long-term conditions/Polypharmacy
	Fitness/Frailty
	Nutritional status/Sarcopenia
	Social ageing (caregiver burden, loneliness)
Allocating resources	Resource availability
	Considering alternative options
	Prioritizing/Triage
Shared decision-making	Sensory impairment and other communication barriers
	Capacity assessment
	Next-of-kin/Surrogate decision maker involvement
	Advance Care Planning, Treatment Escalation Plans

Frailty directly impacts an older adult's autonomy, more specifically, the capacity to make decisions and manage their own life. As frailty levels increase, functional independence and the ability to control one's daily life decline, with this effect becoming more pronounced in the very old, affecting both cognitive and physical functionality [1]. Both factors independently have important implications for ICU admission decision-making.

Particularly in older patients, good communication and meaningful dialogue between the health care team and the patient or the proxy, supplemented by available information from formal or informal advance care planning (ACP), can help individualize goals of care and inform the ICU admission decision. The process of shared decision-making (SDM) is a collaborative process between healthcare professionals and patients/proxies, that aligns with the high value many Western societies ascribe to individual autonomy.

Intensive care is an expensive resource, and consequently, ICU bed availability is limited in many countries [2]. In some high-income countries, the rapidly growing proportion of older patients already challenges ICU resource availability and is predicted to further stress ICU capacity [3]. Other high-income countries currently have sufficient resources to offer ICU admission to the older patients. In this setting, older patients are at increased risk of unwanted variation, namely: (i) over-serving and overly aggressive treatment [4–6], (ii) under-serving on the basis of ageism [7, 8], and (iii) discordant critical care, referring to care that is not in keeping with the older patients’ declared or proxy-stated preferences [9]. It is against this background that we suggest that well-informed SDM ensures that patient autonomy is respected and improves the appropriateness of critical care provided to individual patients, both in terms of its intensity and duration.

There are, however, circumstances in which SDM for ICU admission becomes inappropriate, and the moral principles of autonomy, beneficence, and non-maleficence are subordinated to the ethical principle of justice [10]. In many countries, the day-to-day demand for ICU beds already outstrips supply, and processes are needed to fairly prioritise patients for access to the available resource [11–13]. Even in resource-rich countries, when ICU bed supply could not meet demand at peak times during the recent COVID-19 pandemic, prioritisation processes and triage protocols were developed to ensure fair access to critical care resources [14–16]. In this context, respect for the principle of autonomy must be set aside in favour of the principle of justice and the fair distribution of limited resources.

In this review, we will focus primarily on the role and implementation of SDM during decision-making for older patients acutely referred to intensive care for organ support. We will also address circumstances in which SDM becomes inappropriate and briefly propose alternative approaches.

## Methods

We conducted a non-systematic, narrative review of the literature. A PubMed and Google Scholar search for articles published between January 2006 and March 2026 was performed using the search terms that included combinations of keywords and MeSH terms such as: “Decision Making, Shared”; “Aged, 80 and over”; “Intensive Care”; “Triage”; “Autonomy, Personal”; and “Ageism” using the operators (AND, OR). Additional studies were identified manually from the reference lists and from recently updated clinical practice guidelines and textbook chapters in the field of critical care and medical ethics, relevant to the topic of this review.

Articles were screened by both authors, who prioritized high-impact and foundational publications. Discrepancies in article selection were resolved through consensus among the authors. The information presented in the selected articles was summarized into thematic categories and critically evaluated.

## The use of SDM at the time of ICU admission

### Rationale and justification

SDM in critical care settings has been defined as a collaborative process between health care professionals and patients/proxies aiming to integrate the best available scientific evidence with patients’ wishes and values [17]. SDM is most appropriate when several treatment options are clinically and ethically justified and practically (i.e., legally and in terms of resources) available within a given healthcare context [18]. A wide variety of SDM models have been adopted in clinical practice guidelines [19].

In certain situations, “no escalation or aggressive treatment” is also an acceptable choice. Although included in several SDM guidelines and decision aids, evidence indicates that the “no escalation” option is often under-communicated in SDM, revealing action biases among both patients and healthcare professionals [20].

The benefit of SDM in emergencies and intensive care remains debated. Recent studies have highlighted the need to improve both the extent and quality of SDM in intensive care in general [21] and in older critically ill populations [22, 23]. Becker and coworkers showed that SDM in code status decisions resulted in a significant decrease in full-code orders from 50% to 37.2% and lower decisional uncertainty among participating patients (cluster-randomised trial, Switzerland, N = 2663, median age 68 years) [24]. This contrasts with a prior Cochrane review, which concluded that the effect of SDM on health care professionals’ practice might be more limited [25].

SDM is increasingly integrated into geriatric medicine, where several decision aids have been developed and proven effective in supporting older patients’ healthcare decisions [26]. While these aids can be helpful in ACP, in the acute setting of critical illness and older age, SDM must be tailored to the patient’s individual needs, the high stakes, time constraints, urgency, and the uncertainty and complexity of the situation. Whether and to what extent older patients wish to participate in end-of-life decisions varies widely and is difficult to predict [27, 28]. In addition, local and regional cultural norms may influence SDM procedures in important ways. Despite these constraints, addressing two key questions—“what is the matter with the patient?” and “what matters to the patient?”—lays the foundation for patient-centred care whenever older patients present with acute critical illness.

### Mental capacity assessment

Capacity assessment is the mandatory first step in an SDM process with a critically ill older patient. It is indicated for all patients with mood and/or brain function disturbances that affect decision-making capabilities in at least one of the following four domains: understanding, expressing a choice, appreciation of consequences, and reasoning [29]. Although only a small proportion of critically ill older patients retain decision-making capacity [26], efforts should be made to identify these patients and ensure they are given the opportunity to exercise their right to self-determination. These efforts may include identifying and addressing sensory impairment and other communication barriers, providing pain relief, promoting sleep and reducing the risk of delirium.

Capacity is a medical assessment in relation to a specific decision at a specific point in time (Table 2). A patient may simultaneously have the capacity to make one decision but not another. Several caveats arise when assessing capacity in older critically ill patients:

(i) Capacity in critically ill patients fluctuates substantially, warranting repeated assessment.
(ii) Cognitive impairment does not equate to a lack of capacity in specific domains. Patients living with mild to moderate cognitive impairment may retain the capacity to participate in decisions about treatment goals [30].
(iii) Consciousness does not equal capacity [31].
(iv) An unreasonable patient decision does not preclude capacity. Capacity assessment is independent of the patient and the healthcare provider agreeing on the decision.
(v) A formal capacity assessment should be conducted and documented in all cases when capacity is partial or questionable, when stakes are high and when conflicts or controversies persist. For this purpose, validated tools exist [32, 33].

**Table 2:** Distinctions between Competency and Capacity

	Competency	Capacity
Status	Legal determination made by a judge	Medical assessment of a patient’s decisional ability
Temporality	Static	Situational
Objective	Pertaining to domains of decision-making	Decision-specific
Functionality	Presumed in all adults, but rebuttable	Prerequisite of informed consent

### SDM for the older critically ill patient with retained capacity

In most Western legislatures, patients do not have the right to claim for treatments that are considered medically unsound or ineffective. By contrast, patients may refuse treatment that the medical team strongly recommends, even if it is considered lifesaving. However, the vast majority of SDM in intensive care falls somewhere on a continuum between these extremes, aiming to align treatment intensity with the individual patient’s goals of care.

Older patients report very diverse outcomes of importance to them after critical illness, warranting different levels of care for patients with comparable prognoses. This variation should not be regarded as unfair or reported together with unwanted variation and biases. Several studies highlight that many older patients place a high value on life, making intensive care a desirable option [34, 35]. On the other hand, there is also a large body of evidence from mostly Western countries that older individuals rate permanent dependency a worse outcome than death, especially in the event of reduced cognitive function [36–38].

### SDM for the incapacitated older patient requiring intensive care

There is no clear ethical consensus on how to approach patient autonomy in incapacitated patients with life-threatening illness. Broadly, there are two main approaches to clinical decision-making procedures in the absence of capacity [39]. These approaches are justified because they meet the following ethically informed criteria: (i) the patient’s best interest is achieved or (ii) the patient’s presumed will, as expressed in proxy statements and/or advance directives, is carried out. Decisions based on the patient’s presumed will retain many SDM characteristics, but most philosophers and ethicists agree that the autonomy of incapacitated patients is conceptually distinct from full self-determination by a patient with capacity, as it is bolstered by statements that are distant, either in person (proxy) and/or time (advance directives) from the patient’s actual experience of disease and suffering. In some countries, proxy statements and written advance care plans (living wills) are legally binding, whereas in others they serve varying degrees of advisory function.

### Improving SDM

Decision aids that capture the complexity of an intensive care setting are challenging to develop and validate [40]. Free, online tools are available, for example, the Canadian Critical Care Society provides a comprehensive resource (www.myicuguide.ca) that supports families of ICU patients with well-structured, easily accessible information and communication and decision-making tools [41]. The efficacy of this decision-support tool is currently under study.

Multidisciplinary teamwork is essential when discussing treatment goals with critically ill older patients and their next of kin [42, 43]. Patients’ and their family members' readiness and wish to contribute to or refrain from decision-making should be sought and respected. SDM in intensive care is best understood as a co-creative process among healthcare providers, patients, and their families rather than as a pivotal time point. It encompasses knowledge exchange, negotiating agreements on treatment decisions, managing uncertainty, and a shared responsibility for building a culture of trust. The use of a time-limited trial (TLT) may provide guidance and a formalized procedure that incorporates these elements.

Supporting family decision-makers is essential, as they often find their role stressful and burdensome, with a high proportion experiencing long-term consequences [44]. According to a recent scoping review, SDM studies occasionally describe the cognitive, emotional, and social demands it places on patients and healthcare personnel, while the logistical and financial burdens are rarely addressed [45]. Nevertheless, SDM is generally regarded as beneficial, despite limited knowledge of its costs and potential harms.

### Dealing with the difficulties of prognostication

Accurate prediction of patient prognosis is difficult. A number of predictive scoring systems exist; virtually all incorporate chronological age as a variable but have limitations. Some require little or no laboratory testing, are simple, and can be computed quickly enough to assist immediate decision-making. Examples include the Mortality Probability Admission Model (MPM0) [46], general hospital mortality prediction rule [47], and National Early Warning score (NEWS) [48]. Others require more extensive laboratory results and may take up to 24 h for scoring. More complex scores include the acute physiology and chronic health evaluation (APACHE II) [49], simplified acute physiology score (SAPS II) [50] and sequential organ failure assessment (SOFA) [51], making them unsuitable for informing immediate admission decisions. Simple, easy-to-use scores, such as the clinical frailty scale [52], ASA assessment of functionality [53], and clinical assessment of organ failure, have been proposed for use at the time of presentation for admission [15, 54], but have limited discriminatory performance. Incorporating measures such as the presence or absence of malnutrition, sarcopenia [55] and frailty are particularly attractive for informing prognosis, as they provide estimates of resilience. After considerations such as ensuring score compatibility with the patient's situation, interpreting the score’s calibration and discrimination, and integrating the specific effects of age-related prognostic variability [56], score results in advanced age require further careful clinical and contextual interpretation.

While the prognostic objectivity provided by scoring systems is appealing, overall prognostication by individual doctors has been shown to be roughly equivalent to, and occasionally better than, many of the commonly available scoring systems. In general, ICU doctors are capable of moderately accurate mortality prognostication, especially when consensus among treating doctors is high [57, 58], highlighting the continued current need for informed, but ultimately clinical decision-making. Regardless of the method chosen, when applied to individual patients, substantial prognostic uncertainty remains. Because of the lack of detailed age-specific illness data [59], assessing prognosis in the older population is an even greater challenge [52, 60]. The most pressing need to provide a prognosis exists when prioritisation at admission in the form of resource-mandated triage occurs. In this setting, suggested solutions to enhance accuracy of prediction to make decisions at the time of ICU admission have included the use of more than one, or multiple, scoring systems to inform decisions, or observing responses to therapy over time (with or without the assistance of scoring systems) before making a final decision, the latter being recently described as a TLT [15, 54, 61, 62]. A TLT at the time of admission offers certain advantages in advanced age, both in resource-plentiful and resource-limited settings.

### The time-limited trial (TLT)

A TLT describes a bilateral agreement between the treating team and the patient/proxy to admit the patient to the ICU for treatment (with or without agreed limitations) for a fixed period of time. The treating team keeps the family informed of progress, and, upon the pre-agreed time limit being reached, treatments are withdrawn if therapy is failing or continued if the patient has responded positively to therapy [63]. Throughout the process, the shared decision-making model involving the health care team and the patient/proxy informs decisions about adjusting or stopping treatments.

TLTs may offer some advantages for older patients. Firstly, uncertainty about the likely response to intensive care treatment and the ultimate outcome is common. With sufficient time and observations of the response to ICU therapy, predictions of outcome become more stable, allowing greater consistency and accuracy in prediction [61]. Secondly, at the time of potential ICU admission, patients and/or proxies may be overwhelmed by the circumstances and illness they face, and making rapid, thoughtful decisions is often impossible. A TLT is useful, both to provide more time for the health care team to better assess the patient’s chances of a meaningful recovery, and to provide time to more thoroughly establish a trusting relationship with the patient/proxy, as well as allow the patient/proxy time to better understand the circumstances and vocalise the patient’s wishes and values.

Setting the appropriate time cut-off limit for the trial is challenging. Because of the expected variation in the patient’s conditions and the heterogeneity associated with older age, individualised determinations are likely necessary. While general guidance has been published recommending a range of 3–7 days for hypoxic-ischaemic cerebral injury, end-stage cardiac failure and similar conditions, a longer trial duration, up to 7–14 days, may be needed for conditions such as stroke [63]. Others recommend a limit of 24–72 h for acute conditions, especially in terminal patients [64]. A more recent study, utilising data from the VIP2 study [65], demonstrated that time-dependent patterns of uncertainty that can inform prognostic decisions in response to ICU therapy may last up to 7 days in older critically ill patients [61].

The final consideration is choosing the appropriate outcome measures of TLTs. Outcomes have been categorized into narrowly and broadly defined goals [64]. Narrowly defined goals focus on trends in pathophysiology measures, such as lactate concentrations, organ failure scores, dependence on circulatory support, or failure of weaning efforts, and are suited to acute conditions such as pneumonia, intra-abdominal infections, and septic shock. Broadly defined goals such as wakefulness, responsiveness, mobility, especially as they may predict potential for future independence, are more appropriately applied to patients with stroke, traumatic, or other brain injuries.

If conducted well, TLTs have the potential to reduce the length of ICU stay and improve patient/surrogate satisfaction [66], although there is currently little high-quality outcome-based evidence demonstrating the benefit of time-limited trials compared to routine practice [67].

## When not using SDM is appropriate at the time of ICU admission

### Justifying the diminished role of SDM in resource-mandated triage decisions

Certain values are generally most important to health care professionals. These values include the preservation of human life, the promotion of health, and the effective use of resources while respecting personal autonomy, protecting the vulnerable, and maintaining the therapeutic relationship [10]. However, when resources are limited, accepted healthcare values such as autonomy and fidelity to the individual patient have to be compromised [68]. Establishing which values can be retained and which must be forgone or compromised in the face of limited resources is essential. In the context of resource-mandated triage decision-making in older patients, some key arguments are summarised.

When ICU beds are insufficient to admit all appropriately referred patients who could benefit, even if the benefit is small, some patients must, by necessity, be denied admission because limited resources are allocated to other patients with a greater likelihood of benefit. Furthermore, the declined patients will likely suffer excess morbidity and mortality [69, 70]. Since not all appropriately referred patients can be admitted, prioritisation (triage) must be conducted fairly, in accordance with the moral principle of justice [10].

One might argue that for the individual patient, the fairest method of allocation would be random. This could involve a lottery at the time of referral, or a “first-come, first-served” approach, which might be seen as a form of “natural” lottery. However, this would result in some patients with a very poor chance of benefit from ICU care being admitted, while others with a high chance of benefit would be refused, compromising the efficient use of the ICU. As ICU care can be considered a societal resource in most circumstances, a modified utilitarian ethical approach can guide actions that provide the greatest benefit to the greatest number of individuals in a society [10].

A modified utilitarian approach prioritizes admitting those patients most likely to benefit, resulting in greater overall benefit to society from available resources. Additionally, the consumption of ICU resources required to achieve the anticipated benefit should be estimated, so as to maximize the incremental cost–benefit assessment, with incremental benefit being a comparison with other available alternatives, for example, admission to high-dependency units or the general ward. This approach has been favoured by recent international consensus guidance documents on resource-enforced triage [12, 13]. Although by far the most recommended, it should be acknowledged that this approach is not universally accepted and has previously been criticized by some [71].

It should be stressed that such utilitarian-based triage should be performed on medical grounds only, excluding considerations based on patient age, race, gender, religion, socio-economic status, social standing, or personal beliefs and desires [11]. Importantly, allowing the patient/proxy to influence the triage decision, for example, by assessing the strength of their desire for ICU care, would immediately introduce bias and compromise the key principle on which the process is based, fairness and justice [15, 54]. Thus, the individual patient’s right to autonomy is expressly excluded from the decision-making process, and correspondingly, shared decision-making has no part in influencing the outcome of such decisions.

There is one final consideration regarding the use of shared decision-making following a triage decision. Despite being offered ICU care after triage, some patients, after an appropriate goal-of-care discussion, may elect to exercise their autonomy by declining ICU admission [11, 72]. Sensitivity to the timing of a “goals of care discussion” and to embarking on shared decision-making is required, as such discussions should occur only after a final triage decision to admit/or not is made [11]. This is in line with modified utilitarian triage principles that prioritize distributive justice (and societal good) over autonomy and patient preference.

### The impact of older age on fairness at the time of ICU admission

Geriatricians have for some time recognised that the effect of age on fairness in decision-making is complex and should not be determined solely by chronological age. Importantly, there is abundant evidence that older age adversely affects an individual’s chance of admission to ICU when prioritisation (triage) based on rationing is implemented [68, 69]. However, societal expectations generally hold that the aged are not unfairly prejudiced on the basis of chronological age [72]. Potential solutions to clarify the role of age in triage decision-making, with the goal of enhancing prognostic fairness for older patients at the time of ICU admission rely on the principle that age itself may justify exclusion only indirectly, through objective relationships between age and prognosis [15, 54]. These relationships include the increased organ dysfunction, comorbidities, and functional restrictions that accompany increasing age. When considering ICU admission, care should be taken to ensure that chronological age alone is not a justification for supporting a triage decision. Benefit should be assessed as equitably as possible across all age groups, using an objective combination of markers of prognosis, such as frailty scores, comorbidities, organ function, and pre-admission functionality [15, 54].

### The TLT in ICU resource-restricted settings

In the setting of resource restrictions, a less common and more controversial use of a variation of the traditional TLT is for the treating team to unilaterally set a limit on the duration of treatment for a patient admitted to ICU. The patient/proxy is offered the option of a limited trial of ICU (LT-ICU), with defined limits to the type and/or duration of intensive care treatments provided. If the patient fails to respond to the initially agreed ICU treatments within the agreed timeframe, the treatment team alone may decide to withhold or withdraw further treatment, in accordance with the original agreement. The aim of this LT-ICU is to increase medical certainty, especially in older patients, in whom assessing prognosis is more challenging, while retaining the ability to limit resource consumption. The extra time provided may also allow a more trusting relationship to develop with the patient/proxy and potentially help diffuse perceptions of ageism. As fair distribution of resources is a key decision-making priority, the decision to continue or withdraw therapy at the end of the trial period is not shared but made unilaterally by the treating team [15, 54, 74]. In this specific setting, this approach again aligns with the modified utilitarian principles that prioritize distributive justice over autonomy [74].

### Dealing with futility and potentially inappropriate care at the time of admission

From an ethical and moral perspective, it is generally accepted that ICU care should not be offered to patients who have no realistic possibility of gaining incremental benefit from life-sustaining therapies (e.g., patients who are too sick to benefit, or too well, i.e., the patient is well enough that ICU care is not necessary to ensure a positive outcome). In those patients too sick to benefit, several definitions of poor outcome has been proposed, and differences may exist across regions and among individual practitioners, however one compelling definition states “there is no reasonable expectation that the patient will improve sufficiently to survive outside the acute care setting, or when there is no reasonable expectation that the patient’s neurologic function will improve sufficiently to allow the patient to perceive the benefits of treatment” [75]. This is a specialist medical judgment, and if the treating team judges that ICU admission will not improve outcomes compared with other levels of care, there is no obligation to admit, despite family desires or demands. Multiple professional guidelines support this line of action [11, 75, 76].

Terminology used in this setting varies, with “futile care”, “lack of benefit from care”, and “potentially inappropriate care” being commonly used, although the understanding of these terms differs among authors [76]. Specifically, there is no universal agreement on the definition of futility despite longstanding efforts [75, 77]. Nevertheless, it has been suggested that the predicted absence of a physiological effect or a clear negative benefit-to-harm ratio could be labelled as futility and justify the treatment team in unilaterally withholding ICU admission; however, such circumstances are rare [76]. In most situations, some small benefit, physiological or otherwise, cannot be ruled out with absolute certainty, but ICU care remains very unlikely to yield outcomes relevant to the patient, and will incur costs and potentially inflict harm without creating value. Such “potentially inappropriate care”, after due deliberation and discussion among the treatment team and/or appropriate hospital policy/bodies, and empathic and detailed communication of information with the family/proxy, may still be unilaterally withheld [76], provided no jurisdictional legal restrictions apply.

## Discussion

### Benefits and limitations of family involvement in shared decision making

Older patients may wish to exercise their autonomy through their family members in two ways: (i) the next of kin conveys the patient’s will based on their knowledge of the patient’s treatment preference (i.e. substituted judgment standard); (ii) the next of kin contributes to the decision with broader knowledge of the patient’s personality and values (i.e. best interest standard). Inferring specific treatment preferences from personal values may be difficult without detailed medical knowledge and professional support [78]. Furthermore, the substituted judgment standard has been criticised for low proxy accuracy and a range of empirical biases [28]. Notwithstanding these caveats, most older patients wish for family involvement and place a very high degree of trust in their next of kin, despite the predictive imprecision of proxies. This strong trust seems to be rooted in complex descriptions of human relations, values, and emotions, rather than in a desire to state specific treatment preferences or control the decision process [34, 80].

### Advance planning (ACP) for an unknown future

The preventive ethics approach at the end of life encompasses a wide range of tools and procedures designed to anticipate and address ethical dilemmas before they arise. The idea is to minimize reactive crisis management and offer more proactive, patient-centred treatment. Typical tools include Advance Care Planning (ACP), Advance Directives, Living Wills, Physician Orders for Life-Sustaining Treatments, and Treatment Escalation Plans. Their regulatory anchoring, prevalence, and practical procedures are highly context-dependent, resulting in substantial variation across populations and countries. Although these tools have been promoted and studied for decades, their benefits remain disputed [81]. Recently, efforts have been made to shift ACP away from making decisions for future conditions and instead viewing it as procedures to prepare and empower patients and their next of kin for SDM in future in-the-moment medical decisions [82]. Geriatricians and primary care physicians play an important role in facilitating and coordinating these processes.

### Considerations when shared decision-making is replaced by utilitarian triage

There is a need to set aside the imperatives of beneficence, non-maleficence, and individual autonomy (i.e., self-determination) when resources are critically limited and to act based on principled autonomy (i.e., adherence to a shared understanding of the greater good). Justification for any resource-based refusal of admission to any patient requires that all reasonable efforts to increase the resource (e.g., ICU bed availability) have been exhausted, and that documentation of these efforts be available [11, 15]. Doctors who are forced into triage are regularly and acutely aware that resource-mandated triage decisions are never easy and often emotionally stressful. Patients and their treating healthcare providers who are exposed to triage are likely to find the [11–13] experience equally, if not more challenging. Therefore, it is crucial that ICU admission decision-makers are always honest, fair, consistent, communicate clearly, and, above all, demonstrate empathy and sensitivity. A number of guidelines and consensus statements detailing approaches to implementing resource-mandated triage are available [11–13, 15]. However, only a few directly address the specific challenges of triage in older patients [7, 54].

### Special awareness of the vulnerable

**Fig. 1:**
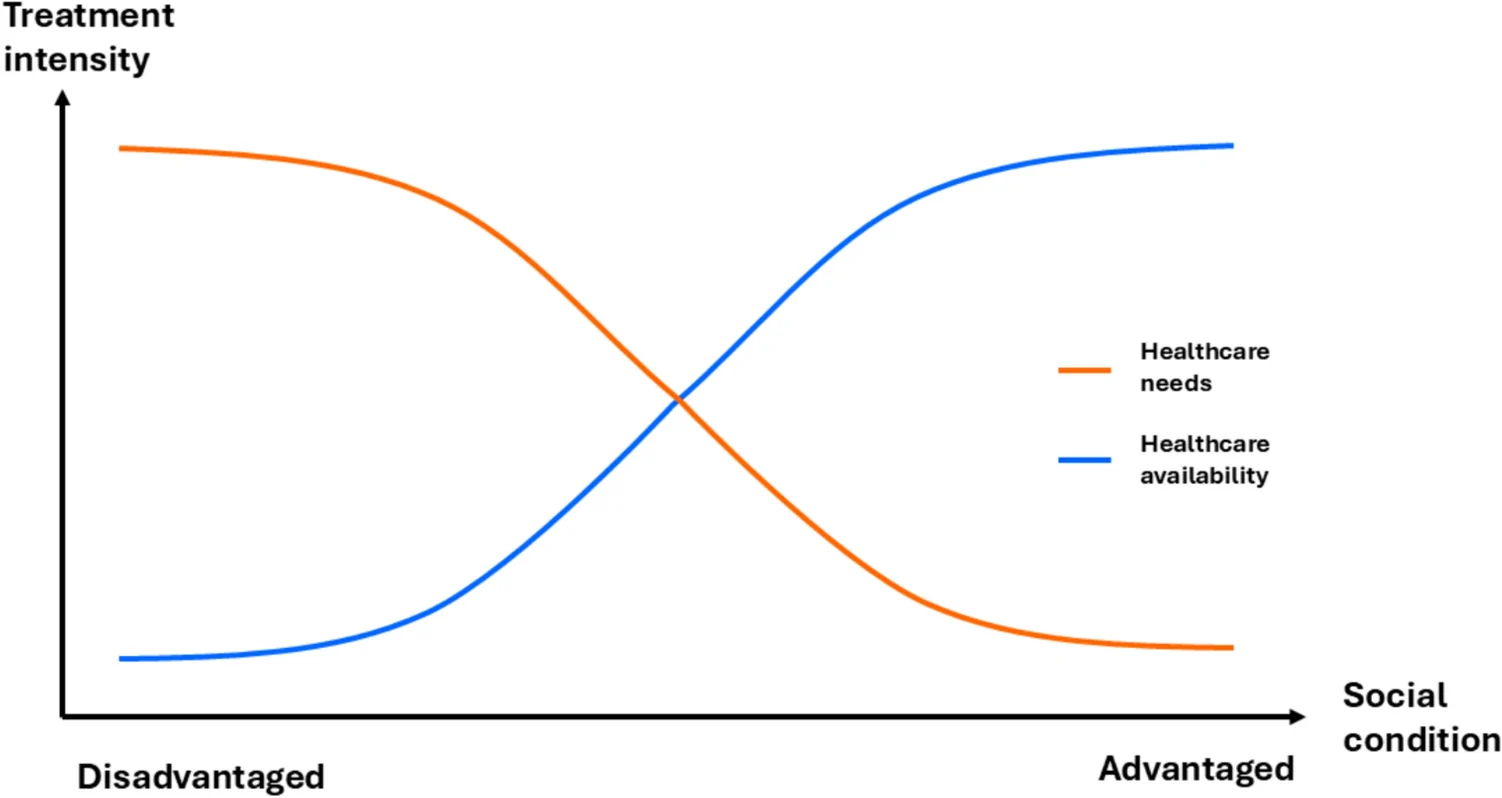
The relationship between healthcare needs and healthcare availability, access and quality in relation to the individual’s social condition

Socio-economically deprived patients and their next-of-kin might not be able to eloquently communicate their preferences and concerns to health care providers with a different social status, a different language, and within very unequal power relations. Additionally, loneliness in older age has been shown to be a major risk factor for adverse health outcomes, both in high- and even more in low-income countries [83]. Therefore, introducing SDM and involving next-of-kin decision-makers as a means of improving the quality of intensive care for older patients carries the inherent risk of worsening inequalities in health care, as conceptualized by the inverse care law [84] (Fig. 1).

### Limitations of rational choice theory in clinical practice

Comprehensive frameworks exist to assist with both shared decision-making [17, 42] and triage [11–13]. They are based on the assumption that these processes are rational choices, whereas there is increasing evidence that these decisions are substantially influenced by social and cultural factors, scientific and personal uncertainty, biases, and emotions. Namely,

(i) vast variability unrelated to patient characteristics or preferences across geographical regions [85], hospitals/units [86], and clinicians [87]. Older age seems to be an important risk factor for such unwanted variation [88].
(ii) increased heterogeneity intrinsic to biological ageing, resulting from the accumulation of a wide range of exposures, experiences, and damage over time. The consequences for ICU admission decisions include reduced discriminatory performance of common prognostic scoring systems [4], likely due to wide variation in chronic long-term conditions that only partially overlap with other prognostic characteristics, e.g., frailty [89].
(iii) complexity of medical uncertainty. Epistemological uncertainty, e.g. prognostic uncertainty that might be reduced by adding new knowledge, is only one dimension, and emotional uncertainty or norm conflicts may contribute to increased decisional uncertainty in older critically ill patients [90, 91].

## Conclusion

Future research priorities should include: (i) increasing the available knowledge of patient-centered short- and long-term outcomes in older ICU patients, such as quality of life as well as psychological, cognitive and physical function. (ii) developing and validating accurate and objective prognostic models based on acute and geriatric patient characteristics, specific for critical illness in advanced age. (iii) describing and evaluating approaches to manage uncertainty and conflicts in the context of life-sustaining intensive care in very old patients [59]. The anticipated future pressure on ICU bed resources, both availability and costs, mandates that research also focuses on establishing objective approaches to low-value and high-value care scenarios. Lastly, we advocate for multidisciplinary research addressing the interplay of medical and social determinants of health and well-being in older adults, which we believe will be of major importance for future intersectoral resource allocation.

To improve the quality of ICU admission decisions in advanced age, we need to foster knowledge of both the rational frameworks designed to support clinical decision-making and the socially constructed nature of these processes. SDM and ACP complement each other in supporting patient-centred clinical decisions. Given correct use and available resources, they lay the foundation for individualized intensive care of older critically ill adults.
